# Antioxidant Capacity and Antimutagenic Potential of *Murraya koenigii*

**DOI:** 10.1155/2013/263509

**Published:** 2013-06-18

**Authors:** Maryam Zahin, Farrukh Aqil, Fohad Mabood Husain, Iqbal Ahmad

**Affiliations:** ^1^Department of Agricultural Microbiology, Aligarh Muslim University, Aligarh 202 002, India; ^2^James Graham Brown Cancer Center, University of Louisville, Louisville, KY 40202, USA

## Abstract

It is well known that the intake of antioxidants with increased consumption of fruits and vegetables and medicinal herbs contributes towards reduced risk of certain diseases including cancers. This study aims to evaluate the broad-spectrum antioxidant and antimutagenic activities as well as to elucidate phytochemical profile of an Indian medicinal plant *Murraya koenigii* (curry) leaves. Leaves of the plant were successively fractionated in various organic solvents. Benzene fraction demonstrated the highest phenolic content followed by petroleum ether. The benzene fraction showed maximum antioxidant activity in all tested assays, namely, phosphomolybdenum, 2,2-diphenyl-1-picrylhydrazyl (DPPH) free radical, ferric reducing antioxidant power (FRAP) and cupric reducing antioxidant capacity (CUPRAC) assays. Based on the promising broad-spectrum antioxidant activity, benzene fraction was further evaluated for antimutagenic activity and showed a dose-dependent antimutagenic response in Ames *Salmonella* mutagenicity assay. It inhibited 72–86% mutagenicity induced by sodium azide, methyl methanesulfonate, benzo(a)pyrene, and 2-aminoflourene at the maximum tested concentration (100 **μ**g/mL) in *Salmonella typhimurium* tester strains. At least 21 compounds were detected by GC/MS. The findings clearly demonstrated that phenolic-rich benzene fraction has promising broad-spectrum antioxidant and antimutagenic property and needs further evaluation to exploit its therapeutic potential.

## 1. Introduction

During the past decades, it became obvious that most degenerative diseases are associated with reactive oxygen species (ROS) such as superoxide anion radicals, hydroxyl radicals, and hydrogen peroxide [1]. Under stress conditions, our bodies produce more ROS which creates homeostatic imbalance and generates oxidative stress and causes cell death and tissue injury [2]. There are endogenous as well as exogenous systems to protect cells from damage in animals. However, in many conditions, the endogenous antioxidants are depleted and therefore exogenous supply of antioxidants becomes essential.

Antioxidants play an important role in biological systems [3] by suppressing the formation of active oxygen species by reducing hydroperoxides (ROO^•^) and H_2_O_2_ and scavenging free radicals among others. Antioxidants are classified as natural/synthetic exogenous or endogenous compounds that can play a role in the removal of free radicals, scavenging ROS or their precursors, inhibiting formation of ROS and binding metal ions needed for catalysis of ROS generation, and so forth [1]. Plant-derived natural products could function as antioxidant especially singlet and triplet oxygen quenchers, peroxide decomposers, enzyme inhibitors, or synergists [4, 5]. 

Human exposure to genotoxic substances present in environment and the food is inevitable. Study of mutagenesis has assumed importance due to the fact that mutations play an important role in carcinogenesis [6]. Consequently, from cancer-preventing point of view, an interest has also been aroused in the presence of antimutagens in foodstuffs as well as in traditionally used medicinal plants, herbs, and spices [7]. On the other hand, mechanism of mutagenesis is complex; however, many mutagens and carcinogens may act through the generation of ROS. Therefore, the discovery and exploration of plant extracts/phytocompounds possessing both antioxidant and antimutagenic properties are of great practical and therapeutic significance. It has also been shown that the increased consumption of fruits and vegetables has generally been found protective against a variety of cancers. Thus, it is presumed that plant with high antioxidant activity could also show antimutagenic activity and counteract the effect of the mutagens and carcinogens [8, 9].

The screening of plant extracts/phytocompounds for antimutagenic activity has been performed using several mutagen assay systems in bacteria, yeast, and some plant and animal cell cultures [10]. Several authors have documented the antimutagenic activity of plant extracts associated with secondary metabolites that act as antimutagen [11–13]. But concerted efforts are needed to explore and exploit the Indian medicinal plants in mutation-related carcinogenesis.

*Murraya koenigii* (family: Rutaceae), commonly known as curry leaf, is a native of India, Sri Lanka, and other south Asian countries and has been used in various cuisine as natural flavouring agent [14]. In addition to its dietary use, this plant has also been used in Indian system of traditional medicine as tonic, stomachic, carminative, and for hypoglycemic properties [15]. The plant has been subjected to intensive investigation and possesses various biological activities [16]. 

We have previously reported that Indian medicinal plants are rich in novel compounds that possess antioxidant and antimutagenic potential [12, 17, 18]. In this paper we discuss the fraction-based antioxidant and antimutagenic properties of curry leaves as well as their active constituents, especially essential oils. The broad-spectrum antioxidant activity using various systems and its antimutagenic potential against both direct and indirect mutagens *in vitro* using Ames *Salmonella* tester strains has also been reported.

## 2. Material and Method

### 2.1. Bacterial Strains and Chemicals

The *Salmonella typhimurium* strains TA97a, TA98, TA100, and TA102 were kindly provided by Professor B. N. Ames, University of California, Berkeley, USA. Sodium azide (NaN_3_), nicotinamide adenine dinucleotide phosphate sodium salt, D-glucose-6-phosphate disodium salt, sodium phosphate, ammonium molybdate, neocuproine, ferric chloride, L-histidine monohydrate, D-biotin and antioxidant standards (butylated hydroxy toluene (BHT), ascorbic acid, and gallic acid) were purchased from Hi-Media Lab. Ltd., Mumbai, India. Potassium ferricyanide, cupric chloride, and ammonium acetate were purchased from Qualigens Fine Chemicals, Mumbai, India. Methyl methanesulfonate (MMS) and trichloroacetic acid were purchased from Sisco-Research Laboratories Pvt. Ltd., Mumbai, India, while 1,1-diphenyl-2-picrylhydrazyl (DPPH) radicals, 2-aminofluorene (2 AF), and benzo(a)pyrene (BP) were purchased from Sigma Chemical Co., St. Louis, MO, USA. All the other reagents used to prepare buffers and media were of analytical grade.

### 2.2. Plant Material and Preparation of Extracts

Leaves of *Murraya koenigii* (L.) were collected locally from the campus of Aligarh Muslim University (AMU), Aligarh, India. The plant was further identified by a taxonomist in the Department of Botany, AMU, Aligarh, and voucher specimen (MBD-14/06) was deposited in the Department of Agricultural Microbiology, AMU, Aligarh, India. The leaves were shade-dried, ground finely and extract was prepared as described earlier [18]. Briefly, five hundred grams of dry leaf powder was soaked in 2.5 L of petroleum ether for 5 days with intermittent shaking and finally, the extract was filtered through Whatman filter paper no. 1 (Whatman Ltd., England) to make a petroleum ether fraction. The collected dried powder was successively extracted with benzene, ethyl acetate, acetone, methanol, and ethanol. The filtered fractions were concentrated to dryness under reduced pressure on rotary evaporator at 40°C and stored at 4°C for future use. The yield of the dried fractions was calculated and extracts were reconstituted in minimum amount of DMSO (≤0.1%) to perform experiments.

### 2.3. Antioxidant Assays

Antioxidant potential of different fractions from curry leaves was determined by DPPH free radical scavenging assay, the ferric reducing power assay (FRAP), and cupric ions (Cu^2+^) reducing ability (CUPRAC) assays as well as total antioxidant capacity was evaluated by phosphomolybdenum method.

#### 2.3.1. Determination of Total Antioxidant Capacity by Phosphomolybdenum Method

The total antioxidant capacity of different fractions was evaluated by the method of Prieto et al. [19]. An aliquot of 0.1 mL of sample solution (containing 12.5, 25, 50, and 100 *μ*g/mL of respective fraction) was combined with 1 mL of reagent (0.6 M sulphuric acid, 28 mM sodium phosphate and 4 mM ammonium molybdate). Methanol (0.1 mL) was used as blank in place of the sample. The tubes were capped and incubated in a boiling water bath at 95°C for 90 min, and then cooled at room temperature. The absorbance of each solution was then measured at 695 nm against a blank in a double beam UV-Vis spectrophotometer UV570455 (EC, Electronic Corporation of India Limited). For samples of unknown composition, water-soluble antioxidant capacity of the extract was expressed as equivalents of ascorbic acid (*μ*mol/g).

#### 2.3.2. DPPH Radical Scavenging Assay

Free radical scavenging activity of different fractions against stable DPPH was determined spectrophotometrically by the modified method of Gyamfi et al. [20] as described earlier [17]. When DPPH reacts with an antioxidant, which can donate hydrogen, it is reduced. The changes in color (from deep violet to light yellow) were measured at 517 nm on a UV-Vis spectrophotometer (Spectronic 20 D+, USA). Fifty microliters of the solvent-dried leaf fractions, yielding 12.5, 25, 50, and 100 *μ*g/mL, respectively, in each reaction was mixed with 1 mL of 0.1 mM DPPH in methanol solution and 450 *μ*L of 50 mM Tris-HCl buffer (pH 7.4). Methanol (50 *μ*L) was used as a vehicle control in the experiment. After 30 min of incubation at room temperature, the reduction of the DPPH free radicals was measured spectrophotometrically. Ascorbic acid and BHT were used as controls. Percent inhibition was calculated from the following equation:

(1) %  inhibition=(a−ba)×100,

where *a* is the absorbance of control and *b* is the absorbance of test sample

#### 2.3.3. FRAP Assay (Fe^3+^ Reducing Power Assay)

The ferric reducing antioxidant power (FRAP) method of Oyaizu [21] with little modification [22] was adopted to measure the reducing capacity. FRAP assay measured direct reduction of Fe^3+^(CN^−^)_6_ to Fe^2+^(CN^−^)_6_, resulting in formation of the Perl's Prussian Blue complex following the addition of excess ferric ions (Fe^3+^). Concentrations of extracts (12.5–100 *μ*g/mL) in 0.75 mL of distilled water were mixed with 1.25 mL of 0.2 M (pH 6.6) sodium phosphate buffer and 1.25 mL of 1% potassium ferricyanide [K_3_Fe (CN_6_)]. After 20 min of incubation at 50°C for 20 min, the reaction mixture was acidified with 1.25 mL of 10% trichloroacetic acid. Finally, 0.5 mL of 0.1% FeCl_3_ was added and absorbance was measured at 700 nm. The increase in absorbance of the reaction mixture indicates greater reduction capability.

#### 2.3.4. CUPRAC Assay

In order to determine the cupric ions (Cu^2+^) reducing ability of curry leaf extracts, the method of Apak et al. [23] was used with a little modification as described by Gülçin [24]. Briefly, 0.25 mL CuCl_2_ solution (0.01 M), 0.25 mL ethanolic neocuproine solution (7.5 × 10^−3^ M), and 0.25 mL CH_3_COONH_4_ buffer (1 M) were added to a test tube, followed by mixing with different concentrations of extracts (12.5–100 *μ*g/mL). The total reaction volume was adjusted to 2 mL with distilled water, and the solution was mixed well. The tubes were stoppered and kept at room temperature for 30 min, and absorbance was measured at 450 nm. Increased absorbance of the reaction mixture indicates increased reduction capability.

### 2.4. Antimutagenicity Assay

The *Salmonella* histidine point mutation assay described by Maron and Ames [25] was used to test the antimutagenic activity of the extracts as described earlier [12, 17]. In the preincubation experiment, a mixture of solvent-dried curry leaves extracts and mutagen, each having a volume of 0.1 mL of varying extract concentrations, was preincubated at 37°C for 30 min and then 0.1 mL of 1 × 10^7^ CFU/mL density of the bacterial culture was added, followed by the addition of 2.5 mL of top agar (0.5% NaCl and 0.6% agar) supplemented with 0.5 mM histidine and biotin each at 45°C. 

The influence of metabolic activation of indirect acting mutagens, BP and 2-AF, was tested using 500 *μ*L of S9 mixture (0.04 mg proteins/mL of mix). The S9 microsome fraction was prepared from the livers of rats treated with Aroclor 1254 using standard protocols [25]. The solution was vortex mixed and poured onto minimal glucose plates (40% glucose and Vogel Bonner medium). The plates were incubated at 37°C for 48 h, after which the numbers of histidine-independent revertant colonies were scored.

Survival of the tester bacterial strains was routinely monitored for each experiment. To check the toxicity of the test samples, parallel controls were run with extracts alone at all concentrations tested with mutagens. The concentrations of the test sample for investigating the antimutagenicity were 12.5, 25, 50, and 100 *μ*g solvent-dried extract/0.1 mL/plate. These were tested against direct acting mutagens sodium azide (1.5 *μ*g/0.1 mL/plate) and MMS (1 *μ*g/0.1 mL/plate) in TA97a, TA98, TA100, and TA102 tester strains as well as against indirect acting mutagens BP and 2-AF. All the test samples and mutagens were dissolved in minimum volume of DMSO. In each case, no toxicity was observed and the numbers of spontaneous revertants were identical to the DMSO vehicle control. Nontoxic concentrations were categorized as those where there was a well-developed lawn, almost similar size of colonies and no statistical difference in the number of spontaneous revertants in test and control plates. Triplicate plates were set up with each concentration and the entire experiment was repeated twice that provided six replicates per treatment. Inhibition of mutagenicity was expressed as percentage decrease of reverse mutation and calculated as:

(2) $$\begin{array}{c} \%\;\text{inhibition}\;=\;\left(a-\frac{b}{a}-c\right)\times 100, \end{array}$$

where *a* is the number of histidine revertants induced by mutagen, *b* is the number of histidine revertants induced by mutagen in the presence of plant extract, and *c* is the number of revertants induced in negative control.

### 2.5. Total Phenolic Content of Plant Fractions

The total phenolic content of the plant fractions was determined using Folin-Ciocalteau reagent by the method in [26], as described earlier [18]. Briefly, to 0.5 mL of each sample (containing 12.5, 25, 50 and 100 *μ*g/mL of the extract), 2.5 mL of 1/10 dilution of Folin-Ciocalteau's reagent and 2 mL of Na_2_CO_3_ (7.5%, w/v) were added and incubated at 45°C for 15 min. Each experiment was performed in triplicate. The absorbance of all samples was measured at 765 nm using a UV-Vis spectrophotometer. Results are expressed as milligrams of gallic acid equivalent per gram of extract dry weight (mg GAE/g dw).

### 2.6. Gas Chromatography Mass Spectrometry (GC-MS)

The active fraction of the extracts was subjected to GC-MS in order to identify the active constituents. The GC was done on GCD 1800 A, Hewlett Packard, coupled with HP-1 column (30 m × 0.25 mm × 0.25 *μ*m; Thermo Scientific, USA). Injector and detector temperatures were 250°C and 280°C, respectively. The carrier gas used was helium at 1 mL/min; initial temperature of oven was 100–250°C at the rate of 10°C/min, hold time was at 250°C for 3 min and final temperature was 250–280°C at the rate of 30°C/min and hold time at 280°C for 2 min. The solvent used for making all dilutions was methanol.

### 2.7. Statistical Analysis

The results are presented as the mean of three experiments with triplicate plates/dose/experiment. The data were further analyzed for statistical significance using analysis of variance (one-way ANOVA) by Tukey test where treatment groups were compared with their respective controls. The regression analysis was carried out in Microsoft Excel 2003 between percent inhibition of mutagenicity and log values of concentrations of the plant extract.

## 3. Results

*Murraya koenigii* has been reported to contain a variety of phytochemicals of medicinal importance. In this study, we analyzed different fractions isolated based on the polarity of the aqueous and organic solvents and compared their relative antioxidant properties. The curry leaves were extracted separately in petrol ether, benzene, ethyl acetate, acetone, methanol and ethanol. Curry leaves yielded 1.0, 1.2, 1.2, 1.8, 2.2, and 0.8 percent of the extract when fractionated in petrol ether, benzene, ethyl acetate, acetone, methanol, and ethanol, respectively.

### 3.1. Antioxidant Activity of Curry

The total antioxidant activity of curry leaves fractions was tested by phosphomolybdenum method and compared with positive controls, ascorbic acid and BHT. All the tested fractions exhibited concentration-dependent antioxidant capacity with respect to ascorbic acid equivalents (Table 1). The benzene fraction showed maximum antioxidant capacity (3510.4 *μ*mol) at 100 *μ*g/mL followed by ethyl acetate (1982.3 *μ*mol), petroleum ether (1967.2 *μ*mol), and acetone (1783.1 *μ*mol) fractions. However, methanol and ethanol fractions displayed relatively less antioxidant capacity (261–639 and 304–369, resp.) at the tested concentrations of 12.5–100 *μ*g/mL.

**Figure 1:**
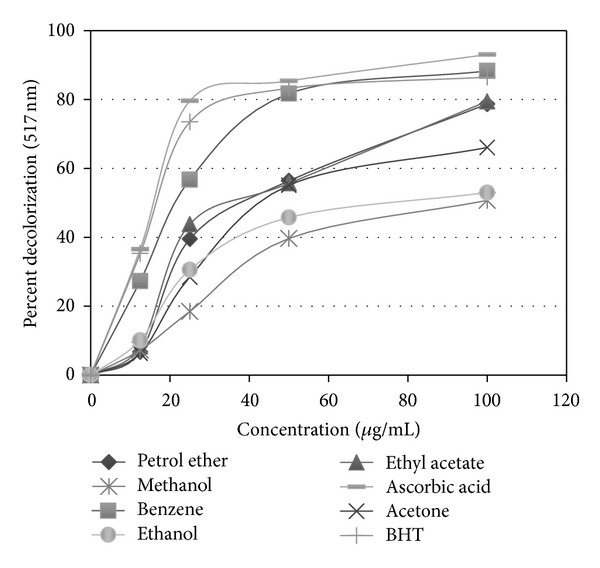
DPPH free radical scavenging activity of *Murraya koenigii *leaf fractions (petroleum ether, benzene, ethyl acetate, acetone, methanol, and ethanol) at different concentrations (12.5–100 *μ*g/mL). The activity is compared with commercial antioxidants, ascorbic acid and BHT. Each data point represents the mean of three experiments. Standard deviation (≤4%) is not shown for clarity of the data.

The free radical scavenging activity was measured as decolorizing activity following the trapping of the unpaired electron of DPPH as shown in Figure 1. The free radical scavenging activity of curry fractions by DPPH method exhibited a concentration-dependent response (Figure 1). The benzene fraction was found to be the most active free radical scavenger exhibited (88.3% decrease at a concentration of 100 *μ*g/mL) followed by ethyl acetate (79.5%) and petrol ether (78.7%) fractions, while positive controls (ascorbic acid and BHT) at a concentration of 100 *μ*g/mL inhibited 93.1% and 86.5% DPPH absorption, respectively. Acetone, methanol, and ethanol fractions showed decolorization of 66.1%, 50.7%, and 53.0%, respectively.

**Figure 2:**
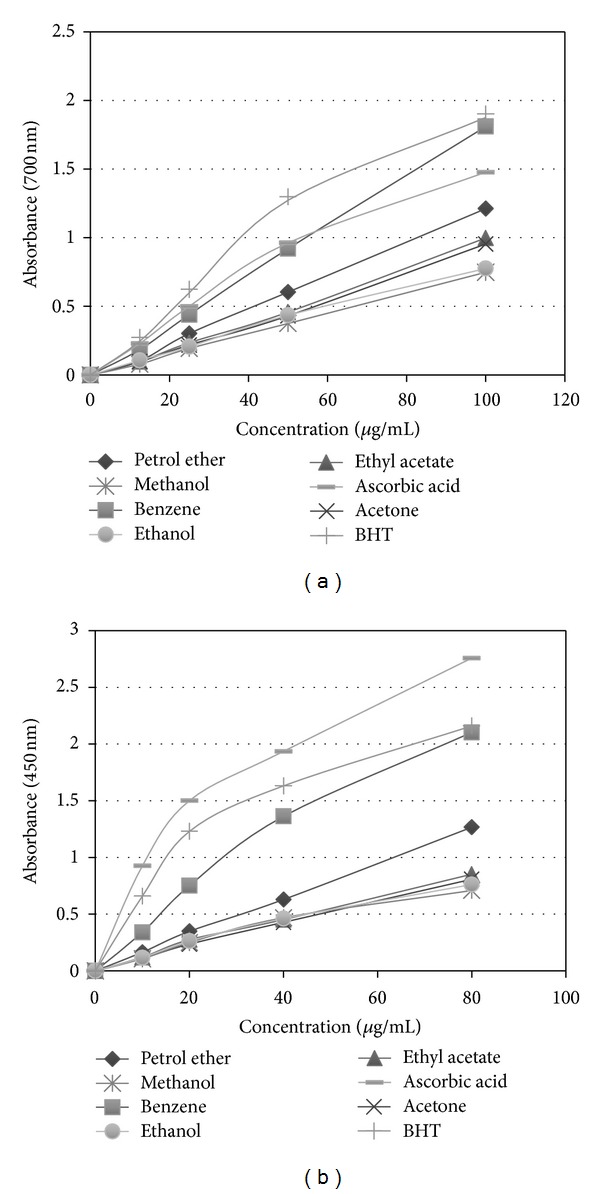
Reducing power of *Murraya koenigii *leaf fractions (petroleum ether, benzene, ethyl acetate, acetone, methanol, and ethanol). The extracts were tested in the range of 10–100 *μ*g/mL concentrations in FRAP (a) and CUPRAC (b) assays and compared with ascorbic acid and BHT. Each data point represents the mean of three experiments. Standard deviation (≤4%) is not shown for clarity of the data.

The FRAP assay has been widely used in the evaluation of antioxidant component in dietary polyphenols. The reducing potential of the curry leaf fractions was concentration-dependent (Figure 2(a)). The benzene fraction demonstrated maximum reducing activity among the fractions. The activity was greater than the ascorbic acid and at par with BHT. The FRAP activity at 100 *μ*g/mL concentration was in the order of BHT = benzene > ascorbic acid > petroleum ether > ethyl acetate > acetone > ethanol, and least in methanol fraction. 

Similarly, the antioxidant activity by CUPRAC assays indicated the highest reducing power potential in benzene fraction followed by petrol ether and ethyl acetate as shown in Figure 2(b). However, ascorbic acid had superior activity compared to benzene fraction. On the other hand, benzene fraction had more CUPRAC activity than BHT.

### 3.2. Antimutagenic Activity

There are several reports suggesting the correlation of antioxidants with high antimutagenic activity. In this study, benzene fraction demonstrating highest antioxidant activity was tested to explore antimutagenic potential of curry. The benzene fraction at concentrations of 12.5, 25, 50 and 100 *μ*g/plate was found nonmutagenic as well as non-toxic to *Salmonella typhimurium *strains, either alone or in the presence of S9 mix. Doses of 1.5 *μ*g/plate of sodium azide and 1 *μ*g/plate of methyl methanesulfonate were chosen for the antimutagenicity study, since these doses were not toxic to the *S. typhimurium* tester strains. Similarly, BP and 2-AF were tested at the concentration of 1 and 5 *μ*g/plate in the presence of S9 mix.

The antimutagenic activity of curry leaves was found to be dose dependent (Tables 2 to 5). At a dose of 100 *μ*g/plate, antimutagenic response was significant (*P* ≤ 0.05) against TA97a with a decrease in the NaN_3_-induced mutagenicity by 84.9% followed by TA100 (84.4%), TA98 (73.2%) and TA102 (72.2%) (Table 2). The linear regression analysis between extract dose and antimutagenic response against respective test mutagen showed strong correlation with respect to dose dependent response ranging from (0.95–1.0), respectively. Similarly, the decrease in number of His^+^ revertants was significant (*P* < 0.005) for *Salmonella typhimurium* TA102 (86.0%) followed by TA100 (83.6%), TA97a (80.0%), and TA98 (74.1%) against MMS-induced mutagenicity as depicted in Table 3. Linear correlation between fraction dose and antimutagenic response was highly significant in all the strains (*R* ^2^ = 0.96 or more). 

The antimutagenicity of *M. koenigii *benzene fraction has also been demonstrated against indirect acting mutagens benzo(a)pyrene (BP) and 2-aminoflourene (2-AF) that infers their mutagenicity by microsomal activation. At 100 *μ*g/plate, the benzene fraction was found antimutagenic for BP-induced mutation at (*P* < 0.001) as evident from the data presented in Table 4. The antimutagenic response was dose-dependent (*R* ^2^ ≥ 0.98), and it ranged from 80.1% to 86.0%. Similarly, trend in antimutagenicity has been shown against 2-AF as presented in Table 5. Further, the linear regression analysis between extract dose and antimutagenic response showed strong correlation ranging from 0.99 to 1.0 against respective tester strains.

### 3.3. Phytochemical Analysis of *Murraya koenigii *(Leaf)

Phytochemical analysis of fractions revealed the presence of alkaloids, phenolics, and glycosides as major group of compounds (data not shown). The total phenolic content as gallic acid equivalent (mg/g) of *Murraya koenigii* fractions showed highest polyphenolic content (187.1  ±  6.3) in benzene fraction followed by petrol ether (146.4  ±  6.3), ethyl acetate (113.9  ±  3.0), acetone (113.9  ±  2.7), ethanol (110.3  ±  2.0), and methanol (103.2  ±  3.1) fractions.

Further, a total of 21 chemical components were identified in the benzene fraction of the leaf extract by GC-MS analysis (Supplementary Figures  1–4; Table S1 in Supplementary Material found online at http://dx.doi.org/10.1155/2013/263509). These numbers may be extended with the help of chemo metric techniques. The major compounds identified were caryophyllene (14.8%) followed by 3-undecen-5-yne (Z)-(9.52%), phytol (9.17%), 2-methyl-3H-phenanthro[3,4-D] imida (8.90%), caryophyllene oxide (6.61%), propylparaben (6.11%), D-limonene (6.01%). The remaining compounds were present in percentages of (1.06–5.72) as depicted in Table 6.

## 4. Discussion

Antioxidants play an important role in biological systems by suppressing the formation of active oxygen species by reducing hydroperoxides (ROO^•^) and H_2_O_2_ and scavenging free radicals among others. Over the past few decades, scientific research has indicated a credible basis for some of the traditional ethnomedicinal uses of spices. Therefore, we have focused our study to elucidate the broad-spectrum antioxidant potential of *M. koenigii* leaf fractions by means of four different *in vitro* tests including total antioxidant capacity by molybdenum method, free radicals scavenging by DPPH, and reducing power by FRAP and CUPRAC assays. The method to detect total antioxidant activity is based on the reduction of Mo (VI) to Mo (V) by the antioxidant compounds with the formation of a green Mo (V) complex which shows maximum absorption at ~700 nm [27]. Whereas other two methods are based on scavenging of free radicals (DPPH) and reduction of Fe[(CN)_6_]_3_ to Fe[(CN)_6_]_2_ in FRAP assay and Cu^2+^ to Cu^1+^ in CUPRAC assay.

This study demonstrated that the sequential fractionation of curry leaves in various solvents resulted in the extraction of antioxidant bioactive compounds mainly in benzene, petrol ether, acetone, methanol and ethanol fractions. The benzene fraction of *M. koenigii* was found to contain the highest total phenolics and to be the most active fraction in all *in vitro* antioxidant assays tested. Other fractions showed activity in order of petrol ether > ethyl acetate > acetone > methanol > ethanol. Polyphenols have been reported to have many beneficial health effects including antioxidant activity. In this study, the total phenolic content was the highest in benzene fraction followed by other suggesting the correlation between antioxidant and polyphenolics content. A series of papers have shown similar correlation including few from this lab [12, 17, 18].

*M. koenigii* leaves were found to have the highest antioxidant activities and total phenolic content in methanol extracts as studied in [28]. These differences might be due to the extraction of curry leaves in methanol only instead of fractionating them into different solvents. Our findings highlight that *M. koenigii* benzene fraction is the potential source of antioxidants. It is probably the first report on the sequential fraction of* M. koenigii* leaves in respective solvents for the evaluation of antioxidant activities. However, other fractions might be having different levels and composition of antioxidant compounds which needs further phytochemical characterization.

Moreover, our results also indicated terpenes as the major components in benzene fraction as identified by GC-MS analysis. It is evident that the antioxidant activities of *M. koenigii* may be related to various carbazole alkaloids and/or phenolic compounds including monoterpenes [29, 30]. Comparative studies of the major components have also indicated terpenes as the most versatile components in* M. koenigii* leaves [29], while many carbazole alkaloids have been isolated and identified from different parts of the plant [31, 32]. Besides these phytochemicals, the curry leaves are also a rich source of nutrients including vitamin A and vitamin C [33].

On the basis of antioxidant potential, the most active fraction (benzene) was tested for its antimutagenic potential against *S. typhimurium* tester strains. Interestingly, a significant antimutagenicity (*P* ≤ 0.05) against direct and indirect acting mutagens in the presence of S9 was recorded. Since BP, a known carcinogenic agent, cannot be metabolized by *Salmonella *to an appreciable extent, so a metabolizing system of supernatant of liver homogenate from Aroclor induced rats (S9) is included in the assay. A number of reactive intermediates like BP-4,5-oxide and 9-hydroxy-BP-4,5-oxide are formed by BP with this activating system that helps in the binding of BP to bacterial DNA [34]. Similarly, the antimutagenic activity against 2-AF-induced mutagenicity was tested in the presence of S9 fraction.

In our study, none of the tested extracts exhibited any mutagenic effect in the Ames test in the absence of enzymatic metabolism. This suggests that DNA does not seem to be a relevant target for the extract tested, and it did not produce DNA lesions that block DNA synthesis, leading to the induction of the SOS system [35]. It is evident from the results of the present study that the antimutagenic behavior of *M. koenigii* is probably due to its antioxidant activities which may be related to various phenolics, monoterpenes and carbazole alkaloids, and so forth [29, 30].

The GC-MS analysis and total phenolic content revealed terpenes as the major active constituents in the tested fraction. The studies on antimutagenic activity of plants have indicated the involvement of chemical constituents which could act as nonspecific redox agents, free radical scavengers, or ligands for binding metals or toxic principles [36]. Besides these, the curry leaves contained carotenoids and retinoids, preformed types of vitamin A, which have been clearly associated with prevention of the induction of cancer and inhibition spontaneous and induced cancers [37]. Recently, terpenes are also reported to have antimutagenic effects in bacterial reverse mutation assay [38] as well as against UV- 4NQO- and t-BOOH induced mutagenesis in *E. coli* K12 and *E. coli* WP2 by reversion assays [39]. Therefore, our study highlights that the benzene fraction of the curry leaf has broad-spectrum antimutagenic properties. Further studies are needed in order to isolate and characterize the active constituents present in it for their therapeutic efficacy.

In summary, we have shown for the first time that curry leaf, yielding highest phenolics in benzene fractions upon sequential fractionation, is a promising source of antioxidant compounds that may be responsible for its antimutagenic properties. Further studies are needed to isolate the active principles, as well as to elucidate the role of various interacting phytocompounds in influencing therapeutic potential and efficacy *in vivo*.

## Supplementary Material

Gas Chromatography Mass Spectrometery (GC-MS): The benzene fraction of the extracts was subjected to GC-MS in order to identify the active constituents. The GC was done on GCD 1800 A, Hewlett Packard, coupled with HP-1 column (30 m × 0.25 mm × 0.25 **μ**m; Thermo Scientific, USA). Injector and detector temperatures were 250°C and 280°C, respectively. The carrier gas used was helium at 1 mL/min; initial temperature of oven was 100–250°C at the rate of 10°C/min, hold time was at 250°C for 3 min and final temperature was 250–280°C at the rate of 30°C/min and hold time at 280°C for 2 min. The solvent used for making all dilutions was methanol.
A total of 21 chemical components were identified in leaf extract by GC-MS analysis (Figure S1). These numbers may be extended with the help of chemo metric techniques. The major compounds identified were caryophyllene (14.8%) followed by 3-undecen-5-yne (Z)-(9.52%), phytol (9.17%), 2-methyl-3H-phenanthro[3,4-D] imida (8.90%), caryophyllene oxide (6.61%), propylparaben (6.11%), D-limonene (6.01%). The remaining compounds were present in percentages of (1.06-5.72) as depicted in Figure S2, S3 and S4 and table S1. Figures S1-S4 demonstrate the mass spectrometric analysis of major 12 peaks demonstrated in Table S1.

## Figures and Tables

**Table 1:** Antioxidant capacity of *Murraya koenigii* leaf fractions expressed as ascorbic acid equivalents (*µ*mol/g of extract) by phosphomolybdenum method.

Concentration (*µ*g/mL)	Antioxidant capacity (*µ*mol/g of extract)					
	Petrol ether	Benzene	Ethyl acetate	Acetone	Methanol	Ethanol
12.5	980.0^d^ ± 18.3	1669.8^d^ ± 21.1	939.4^d^ ± 43.4	702.4^d^ ± 26.2	261.0^d^ ± 15.8	303.7^d^ ± 13.4
25	1531.6^c^ ± 50.7	2459.5^c^ ± 75.4	1399.2^c^ ± 19.5	1233.8^c^ ± 47.1	398.5^c^ ± 21.2	419.3^c^ ± 10.1
50	1663.0^b^ ± 56.9	3132.3^b^ ± 97.2	1589.2^b^ ± 66.9	1438.5^b^ ± 39.4	522.5^b^ ± 12.6	542.3^b^ ± 16.6
100	1967.2^a^ ± 67.4	3510.4^a^ ± 95.8	1982.3^a^ ± 84.3	1783.0^a^ ± 86.4	638.7^a^ ± 25.1	668.8^a^ ± 15.3

LSD at 5%	109.01	116.04	102.06	81.02	43.89	7.93

The above data are the mean of three experiments ± SD; different letters in columns show significant difference in means. LSD: least significance difference.

**Table 2:** Effect of benzene fraction of *Murraya koenigii *on the mutagenicity induced by sodium azide (NaN_3_) using *Salmonella typhimurium* strains.

Treatment	Dose (*µ*g/plate)	Number of His^+^ revertants colonies/plate			
		TA97a	TA98	TA100	TA102
Spontaneous		140.0 ± 4.0	31.0 ± 3.6	128.7 ± 9.8	245.7 ± 23.3
Positive control (NaN_3_)	1.5	246.3 ± 16.0	52.7 ± 2.1	352.0 ± 33.0	343.3 ± 33.3
^a^ *Murraya koenigii *	12.5	164.3 ± 12.3	46.0 ± 4.0	191.7 ± 13.3	286.7 ± 26.3
	25	145.0 ± 15.5	37.3 ± 7.0	172.3 ± 18.9	260.0 ± 24.1
	50	124.7 ± 9.1	30.7 ± 8.5	153.0 ± 19.7	245.3 ± 24.4
	100	118.0 ± 10.5	24.0 ± 4.6	127.0 ± 15.5	228.3 ± 19.6
^b^ *Murraya koenigii +* NaN_3_	12.5	225.3 ± 33.6 (25.6)	51.3 ± 6.0 (20.0)	316.3 ± 32.0 (22.3)	332.3 ± 34.9 (19.4)
	25	200.7 ± 22.7* (45.1)	45.3 ± 4.2 (47.8)	258.0 ± 29.6* (52.3)	313.0 ± 32.1 (36.4)
	50	168.0 ± 22.3** (64.4)	39.0 ± 8.2* (62.1)	212.7 ± 26.6** (70.0)	292.7 ± 18.6 (51.7)
	100	137.3 ± 12.7*** (84.9)	31.7 ± 6.5** (73.2)	162.0 ± 21.0** (84.4)	260.3 ± 14.0* (72.2)

*F* value		18.069	8.288	33.690	6.917

*R* ^2^ value		0.999	0.953	0.969	0.996

The data represented in the table is the mean ± SD values of three replicates. Mean values followed by asterisk are significantly different when compared to positive control at *P < 0.05, **P < 0.005, and ***P < 0.001. The values in parentheses are the inhibition rates of mutagenicity (%). Positive control: NaN_3_, sodium azide. ^a^Negative control; ^b^preincubation test; *R* ^2^: linear regression analysis.

**Table 3:** Effect of benzene fraction of *Murraya koenigii *on the mutagenicity induced by methyl methanesulfonate (MMS) using *Salmonella typhimurium* strains.

Treatment	Dose (*µ*g/plate)	Number of His^+^ revertants colonies/plate			
		TA97a	TA98	TA100	TA102
Spontaneous		140.0 ± 4.0	31.0 ± 3.6	128.7 ± 9.8	245.7 ± 23.3
Positive control (MMS)	1	443.7 ± 19.9	51.0 ± 2.7	952.7 ± 46.0	1195.3 ± 79.0
^a^ *Murraya koenigii *	12.5	164.3 ± 12.3	46.0 ± 4.0	191.7 ± 13.3	286.7 ± 26.3
	25	145.0 ± 15.5	37.3 ± 7.0	172.3 ± 18.9	260.0 ± 24.1
	50	124.7 ± 9.1	30.7 ± 8.5	153.0 ± 19.7	245.3 ± 24.4
	100	118.0 ± 10.5	24.0 ± 4.6	127.0 ± 15.5	228.3 ± 19.6
^b^ *Mu* *rr* *ay* *a* *koenigii*+ MMS	12.5	388.3 ± 31.2 (19.8)	50.3 ± 4.0 (13.4)	802.3 ± 57.1* (19.8)	862.3 ± 58.8** (36.6)
	25	309.0 ± 30.8* (45.1)	45.0 ± 4.6 (43.9)	565.0 ± 45.1*** (49.7)	675.3 ± 56.9*** (55.6)
	50	239.7 ± 20.2** (63.9)	38.3 ± 4.0* (62.3)	440.7 ± 48.6*** (64.0)	518.0 ± 32.1*** (71.3)
	100	183.0 ± 17.5*** (80.0)	31.0 ± 5.6** (74.1)	262.3 ± 31.7*** (83.6)	363.7 ± 37.1*** (86.0)

*F* value treatment		81.635	9.877	225.881	178.255

*R* ^2^ value		0.989	0.958	0.978	0.996

The data represented in the table is the mean ± SD values of three replicates. Mean values followed by asterisk are significantly different when compared to positive control at *P < 0.05; **P < 0.005 and ***P < 0.001. The values in parentheses are the inhibition rates of mutagenicity (%). Positive control: MMS: methyl methanesulfonate. ^a^Negative control; ^b^Pre-incubation; *R* ^2^: Linear regression analysis.

**Table 4:** Effect of benzene fraction of *Murraya koenigii *on the mutagenicity induced by benzo(a)pyrene with metabolic activation using *Salmonella typhimurium* strains.

Treatment	Dose (*µ*g/plate)	Number of His^+^ revertants colonies/plate			
		TA97a	TA98	TA100	TA102
Spontaneous		143.7 ± 13.2	37.0 ± 3.0	135.3 ± 9.5	316.0 ± 14.5
Positive control (BP)	1	720.3 ± 28.5	155.0 ± 7.6	695.7 ± 17.0	648.0 ± 17.1
^a^ *Mu* *rr* *ay* *a* *koenigii *	12.5	142.0 ± 18.3	50.3 ± 2.5	132.7 ± 16.0	265.0 ± 28.2
	25	156.0 ± 14.4	42.7 ± 3.5	155.3 ± 17.2	296.3 ± 17.6
	50	168.7 ± 21.7	35.0 ± 3.6	168.0 ± 18.0	322.7 ± 31.1
	100	181.3 ± 27.1	28.0 ± 3.6	175.3 ± 20.6	340.3 ± 31.3
^b^ *Mu* *rr* *ay* *a* *koenigii+* BP	12.5	592.3 ± 44.5* (22.1)	132.7 ± 19.2 (21.3)	540.0 ± 50.1** (27.7)	560.3 ± 31.2* (22.9)
	25	501.7 ± 36.6** (38.7)	101.0 ± 19.3* (48.1)	435.3 ± 46.1*** (48.2)	501.7 ± 16.9*** (41.9)
	50	382.7 ± 35.6*** (61.2)	80.7 ± 12.7*** (61.9)	352.3 ± 30.7*** (65.1)	436.0 ± 38.2*** (65.2)
	100	276.0 ± 26.5*** (82.4)	53.3 ± 13.9*** (80.1)	253.7 ± 21.4*** (84.9)	382.0 ± 29.2*** (86.0)

*F* value treatment		168.149	49.036	149.651	67.488

*R* ^2^ value		0.996	0.982	0.999	0.999

The data represented in the table is the mean ± SD values of three replicates. Mean values followed by asterisk are significantly different when compared to positive control at *P < 0.05; **P < 0.005 and ***P < 0.001. The values in parentheses are the inhibition rates of mutagenicity (%). Positive control: BP: benzo(a)pyrene.^a^Negative control; ^b^Pre-incubation test; *R* ^2^: Linear regression analysis.

**Table 5:** Effect of benzene fraction of *Murraya koenigii *on the mutagenicity induced by 2-aminofluorene (2-AF) with metabolic activation using *Salmonella typhimurium* strains.

Treatment	Dose (*µ*g/plate)	Number of His^+^ revertants colonies/plate			
		TA97a	TA98	TA100	TA102
Spontaneous		143.7 ± 13.2	37.0 ± 3.0	135.3 ± 9.5	316.0 ± 14.5
Positive control (2AF)	5	328.0 ± 12.0	262.3 ± 17.2	502.0 ± 19.1	1430.3 ± 32.5
^a^ *Murraya * *koenigii *	12.5	142.0 ± 18.3	50.3 ± 2.5	132.7 ± 16.0	265.0 ± 28.2
	25	156.0 ± 14.4	42.7 ± 3.5	155.3 ± 17.2	296.3 ± 17.6
	50	168.7 ± 21.7	35.0 ± 3.6	168.0 ± 18.0	322.7 ± 31.1
	100	181.3 ± 27.1	28.0 ± 3.6	175.3 ± 20.6	340.3 ± 31.3
^b^ *Murraya koenigii*+ 2-AF	12.5	276.3 ± 31.1 (27.8)	200.7 ± 17.0* (29.1)	422.3 ± 32.7* (21.6)	1185.3 ± 77.0** (21.0)
	25	248.7 ± 28.0* (46.1)	161.0 ± 12.5** (46.1)	346.3 ± 21.6*** (44.9)	960.7 ± 70.8*** (41.4)
	50	220.0 ± 25.0** (67.8)	114.7 ± 13.1*** (65.0)	280.7 ± 27.0*** (66.3)	655.0 ± 55.3*** (70.0)
	100	202.3 ± 14.2*** (85.7)	70.0 ± 7.6*** (82.1)	205.0 ± 18.0*** (90.9)	462.0 ± 32.9*** (88.8)

*F* value treatment		24.643	197.239	37.820	276.179

*R* ^2^ value		0.987	0.999	0.999	0.994

The data represented in the table is the mean ± SD values of three replicates. Mean values followed by asterisk are significantly different when compared to positive control at *P < 0.05; **P < 0.005 and ***P < 0.001. The values in parentheses are the inhibition rates of mutagenicity (%). Positive control: 2-AF, 2-aminoflourene.^a^Negative control; ^b^Pre-incubation test; *R* ^2^: Linear regression analysis.

**Table 6:** Components of *Murraya koenigii* benzene fraction as identified by GC-MS analysis.

Peak no.	Components	Retention time	Area (%)
(1)	Caryophyllene	11.19	14.8
(2)	*α*-Caryophyllene	11.77	2.79
(3)	1H-Cyclopropa[a] naphthalene, 1a,2,	12.37	2.93
(4)	Caryophyllene oxide	13.24	6.61
(5)	Spiro[4.4]nonan-2-one	13.60	2.49
(6)	10,10-Dimethyl-2,6-dimethylenebicy	13.93	3.32
(7)	D-Limonene	14.10	6.01
(8)	Propylparaben	15.80	6.11
(9)	Salicylamide	16.00	1.42
(10)	9-Borabicyclo[3.3.1]nonane, 9-hydr	18.55	5.72
(11)	1,2-Diphenylethanethione	18.69	0.54
(12)	Phytol	18.91	9.17
(13)	11,14-Eicosadienoic acid, methyl e	19.44	2.59
(14)	3-Undecen-5-yne, (Z)-	19.58	9.52
(15)	Phenol, 2-methoxy-4-[2-(4-hydroxyphenyl)]	20.71	1.94
(16)	2,6-Octadien-1-ol, 3,7-dimethyl-,	22.41	1.06
(17)	Docosane, 11-butyl-	23.95	5.30
(18)	2-Methyl-3H-phenanthro[3,4-D] imida	24.02	8.90
(19)	Propanenitrile	24.52	2.21
(20)	Pentatriacontane	25.82	4.22
(21)	Vitamin A aldehyde	26.57	2.33
