# Supplementary material for: Antioxidant Capacity and Antimutagenic Potential of Murraya koenigii
Source: Biomed Res Int. 2013 Jun 18;2013:263509. doi: 10.1155/2013/263509 (PMC3703397; doi:10.1155/2013/263509)
Supplement: Supplementary file 1 — Gas Chromatography Mass Spectrometery (GC-MS): The benzene fraction of the extracts was subjected to GC-MS in order to identify the active constituents. The GC was done on GCD 1800 A, Hewlett Packard, coupled with HP-1 column (30 m × 0.25 mm × 0.25 μm; Thermo Scientific, USA). Injector and detector temperatures were 250°C and 280°C, respectively. The carrier gas used was helium at 1 mL/min; initial temperature of oven was 100–250°C at the rate of 10°C/min, hold time was at 250°C for 3 min and final temperature was 250–280°C at the rate of 30°C/min and hold time at 280°C for 2 min. The solvent used for making all dilutions was methanol. A total of 21 chemical components were identified in leaf extract by GC-MS analysis (Figure S1). These numbers may be extended with the help of chemo metric techniques. The major compounds identified were caryophyllene (14.8%) followed by 3-undecen-5-yne (Z)-(9.52%), phytol (9.17%), 2-methyl-3H-phenanthro[3,4-D] imida (8.90%), caryophyllene oxide (6.61%), propylparaben (6.11%), D-limonene (6.01%). The remaining compounds were present in percentages of (1.06-5.72) as depicted in Figure S2, S3 and S4 and table S1. Figures S1-S4 demonstrate the mass spectrometric analysis of major 12 peaks demonstrated in Table S1. [file 263509.f1.docx]

**Supplementary figures and table**

**Fig S1.** GC-MS analysis of *Murraya koenigii* benzene fraction


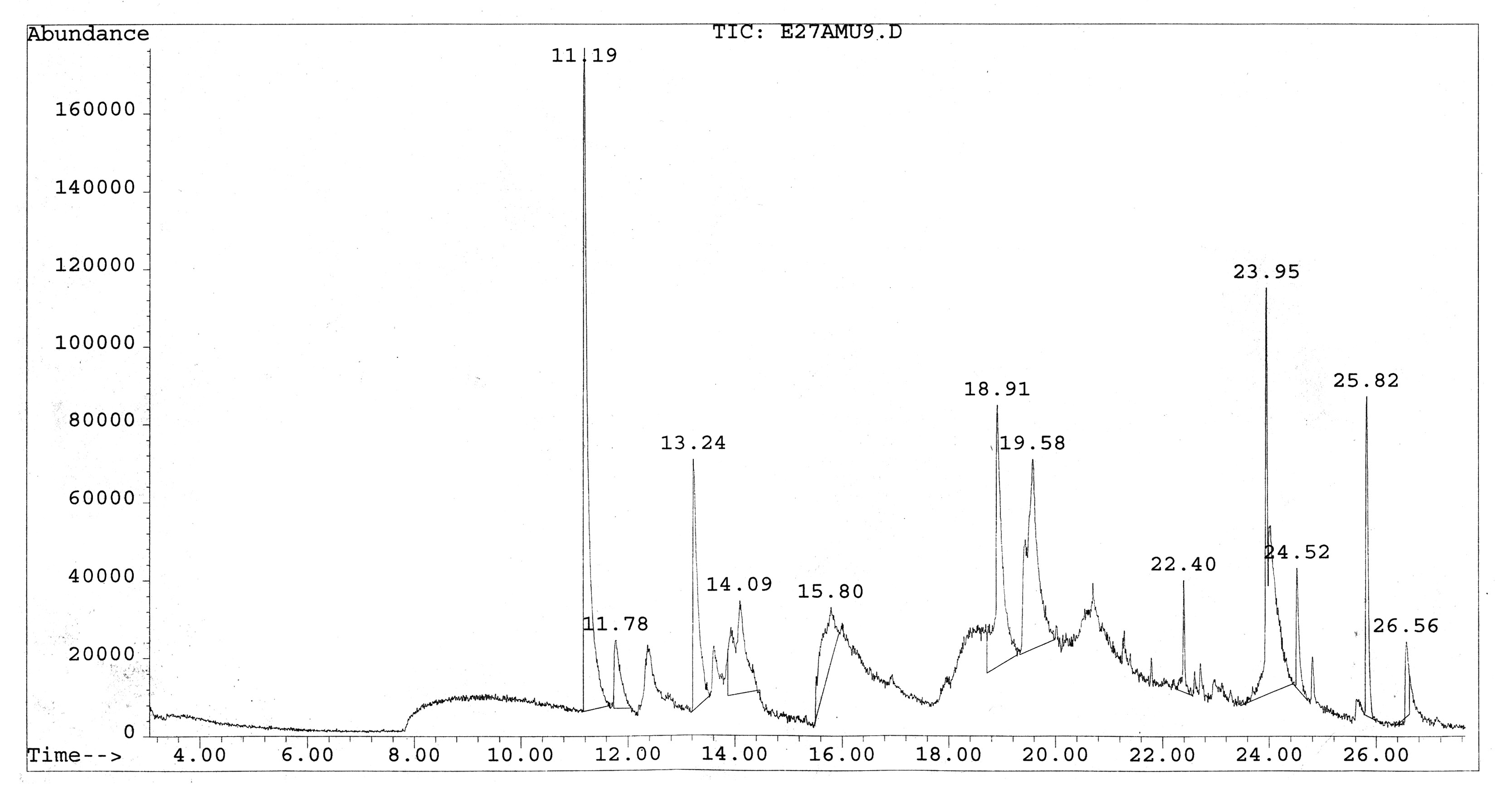


**Fig S2.** GC-MS analysis of *Murraya koenigii* benzene fraction

**
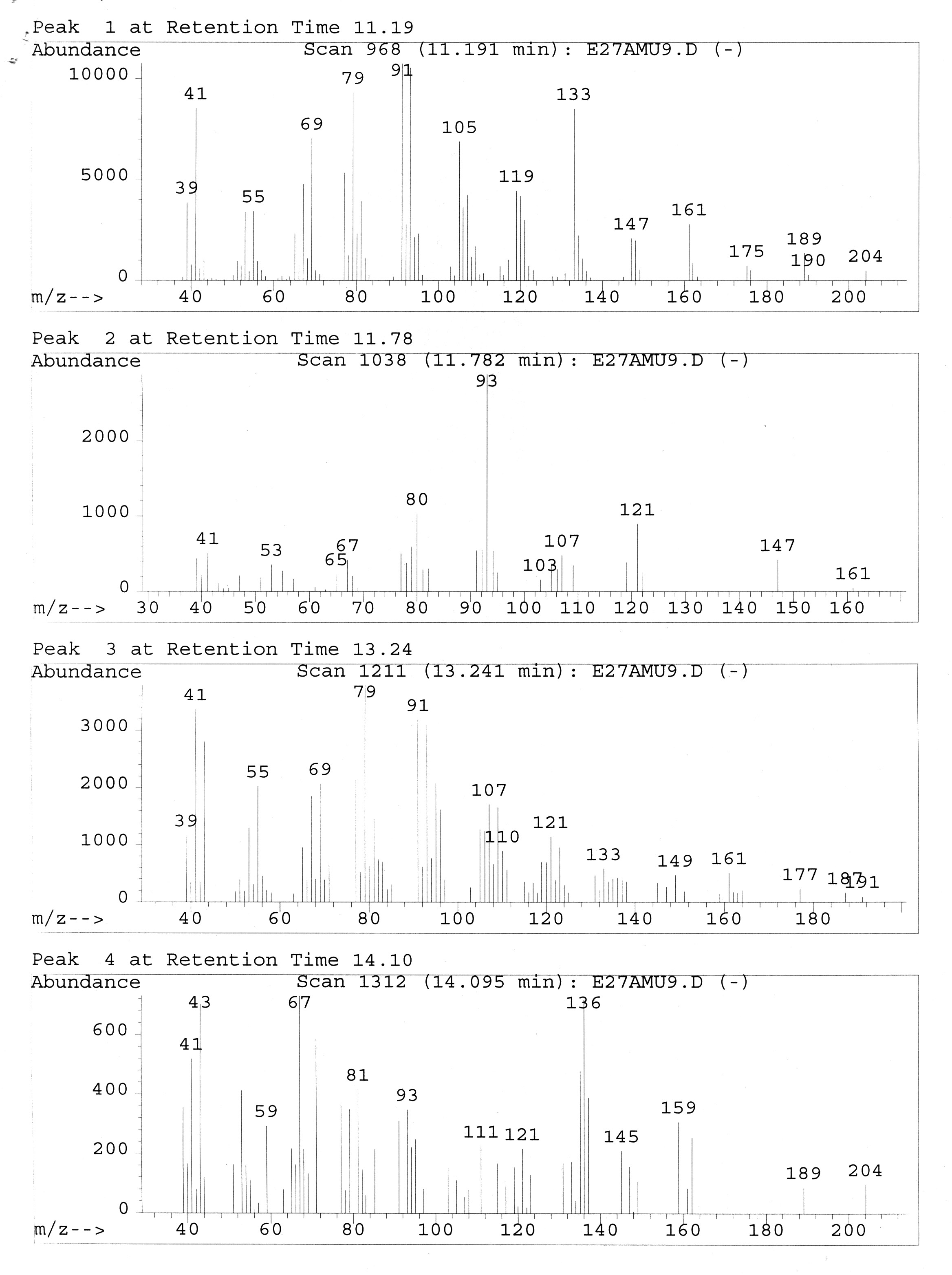
**

**Fig S3.** GC-MS analysis of *Murraya koenigii* benzene fraction

**
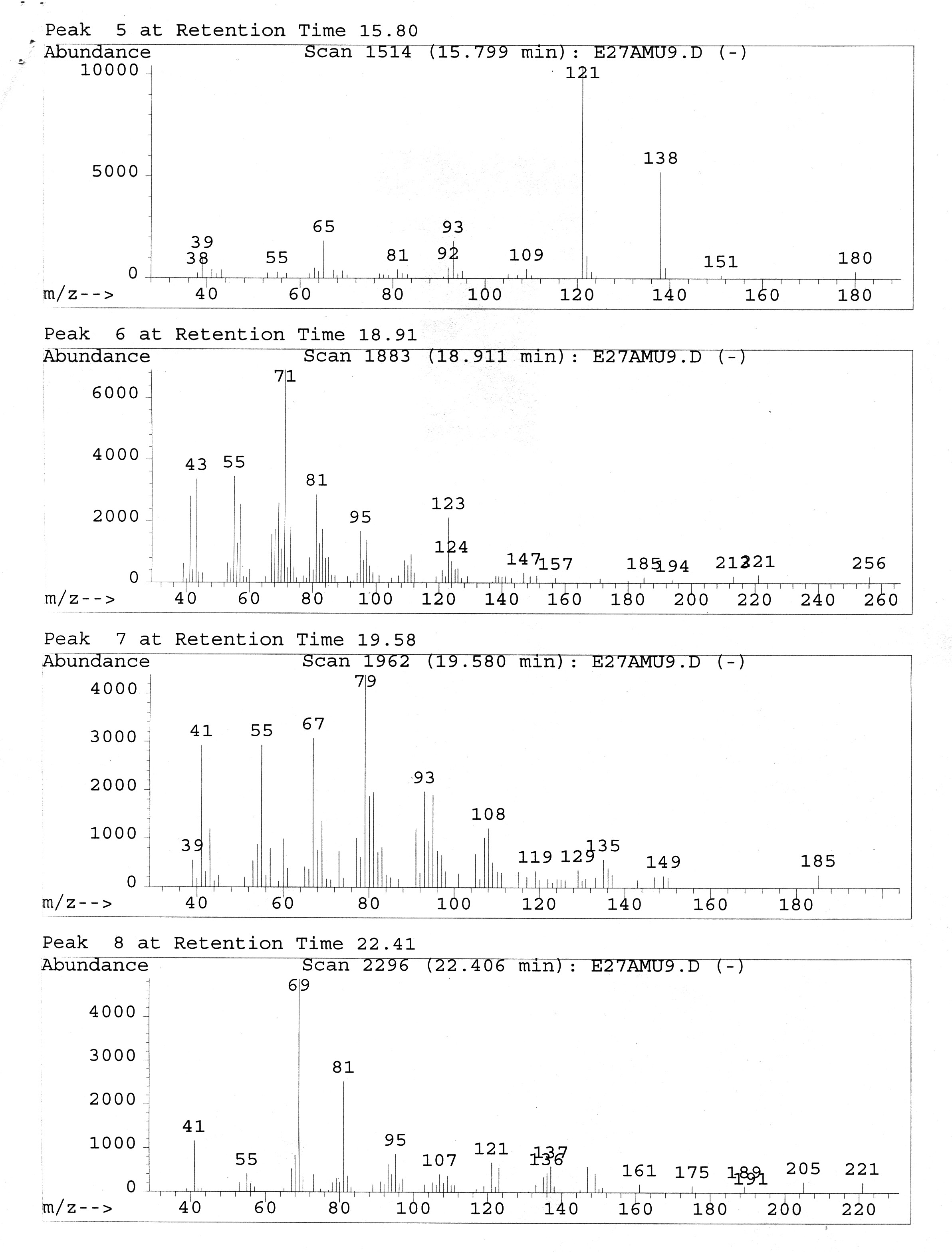
**

**Fig S4.** GC-MS analysis of *Murraya koenigii* benzene fraction

**
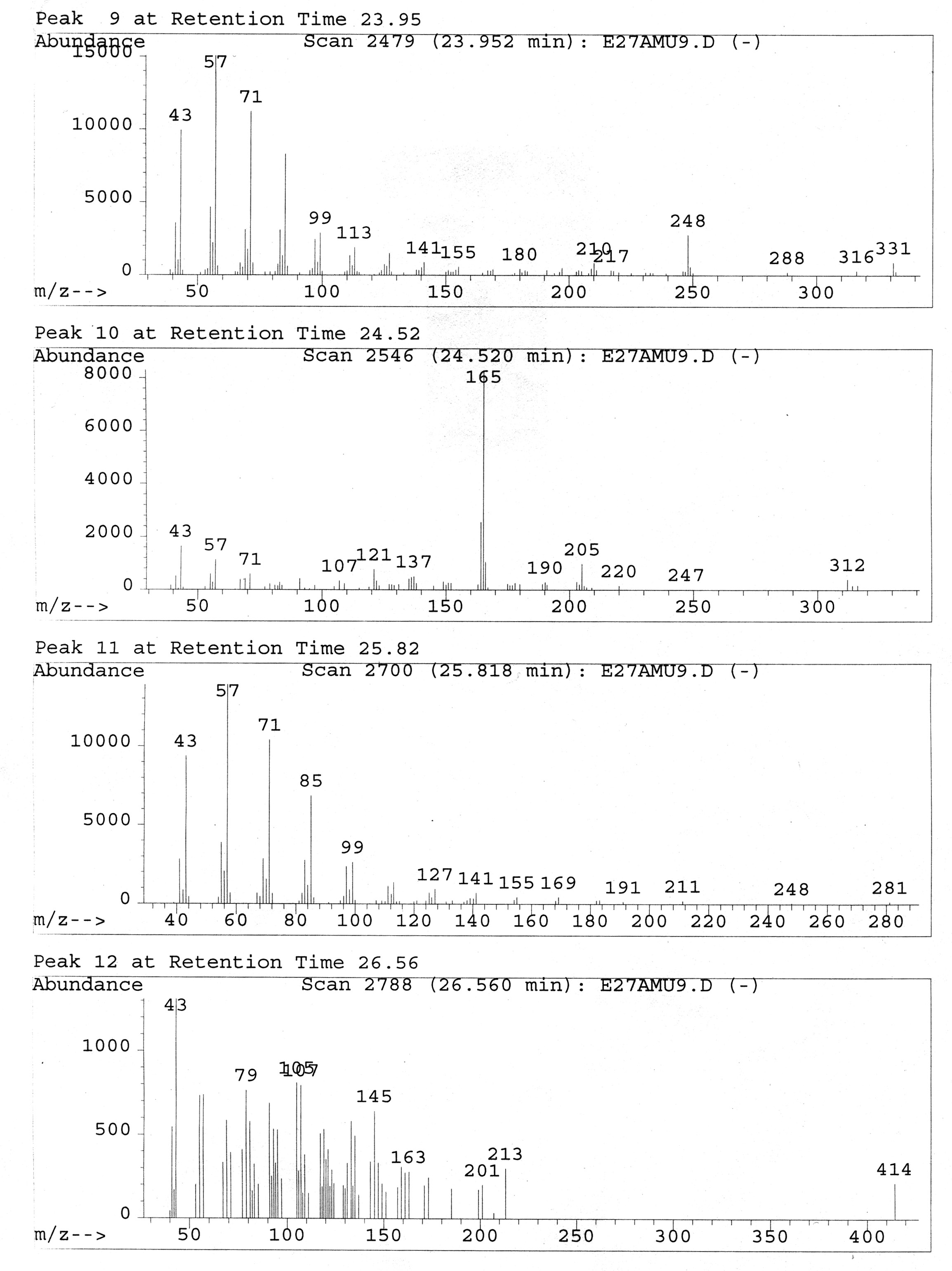
**

**Table S1.** Chemical composition of of *Murraya koenigii* benzene fraction by GC-MS analysis


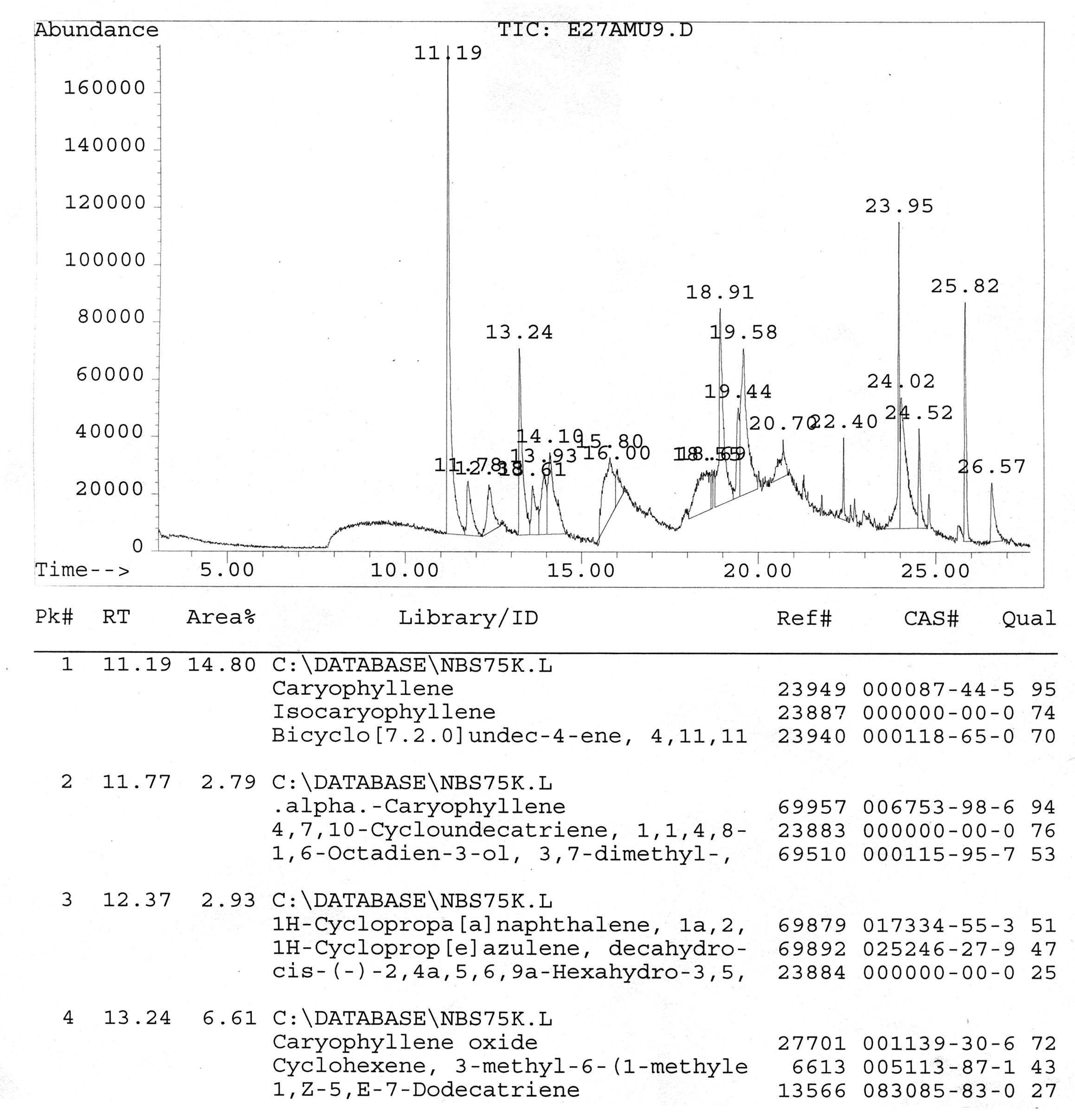


**
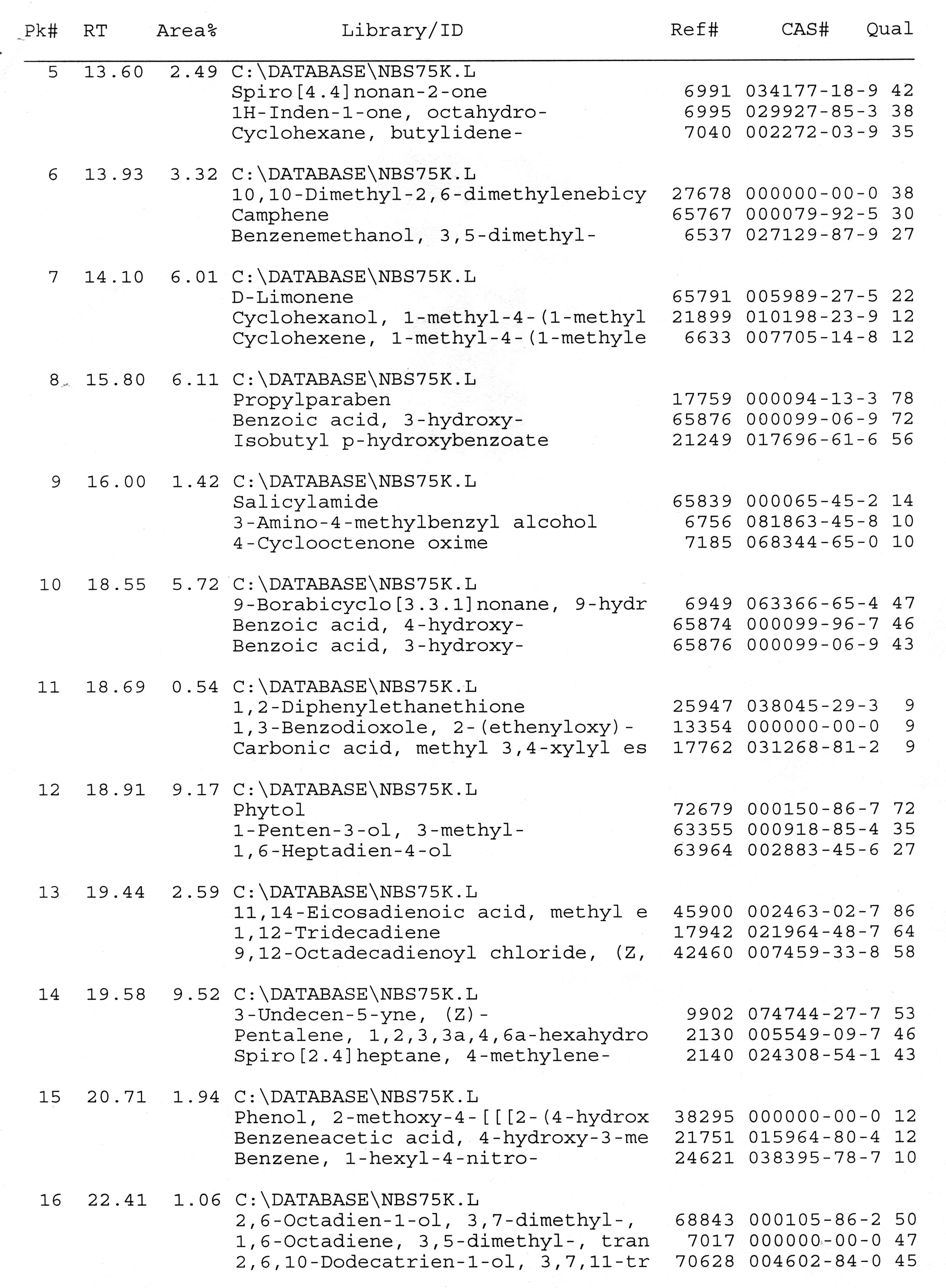
**

**
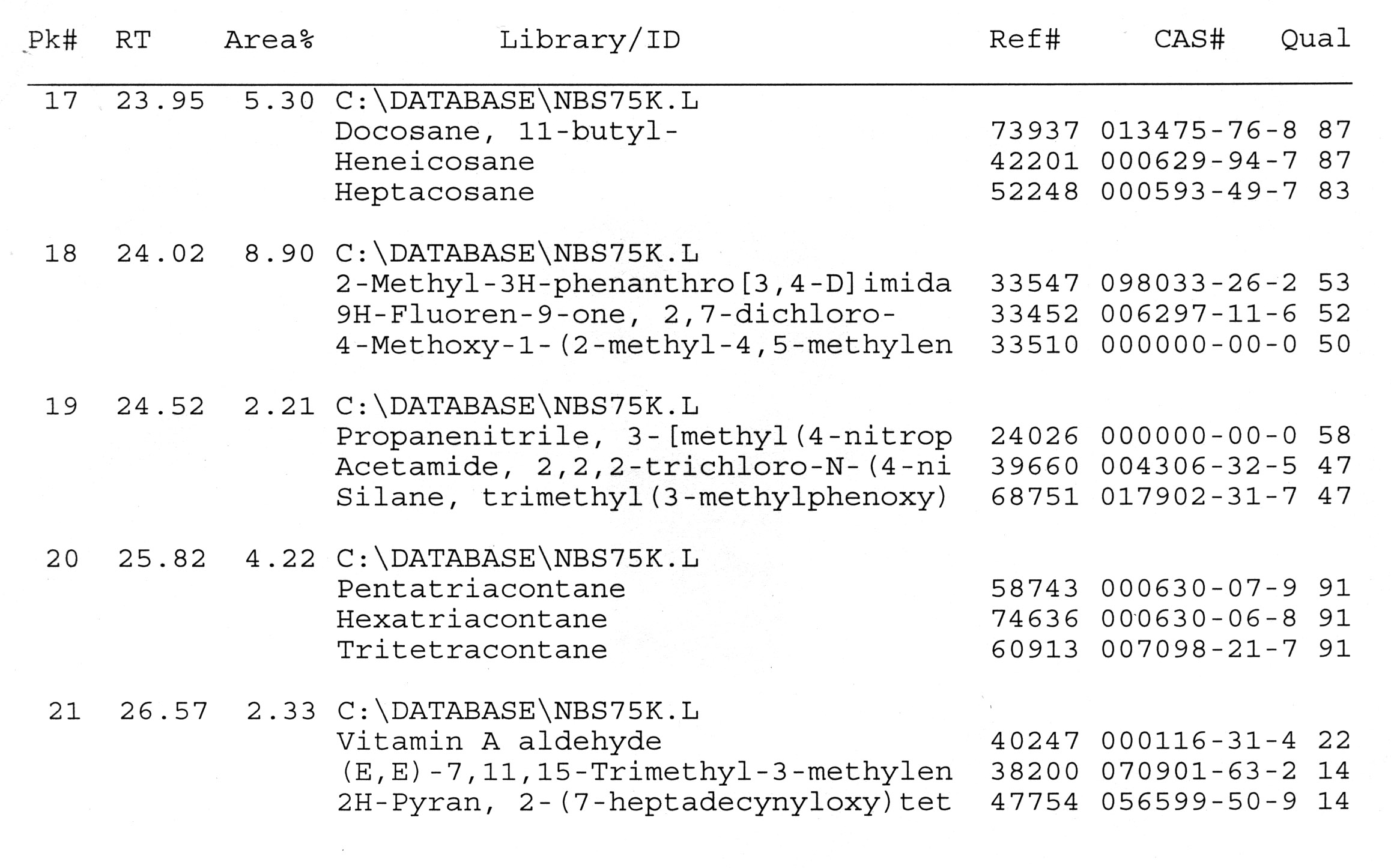
**
